# Constitutive activation of the DNA damage response pathway as a novel therapeutic target in diffuse large B-cell lymphoma

**DOI:** 10.18632/oncotarget.2720

**Published:** 2015-01-07

**Authors:** Enrico Derenzini, Claudio Agostinelli, Enrica Imbrogno, Ilaria Iacobucci, Beatrice Casadei, Elisa Brighenti, Simona Righi, Fabio Fuligni, Andrea Ghelli Luserna Di Rorà, Anna Ferrari, Giovanni Martinelli, Stefano Pileri, Pier Luigi Zinzani

**Affiliations:** ^1^ Institute of Hematology and Medical Oncology L.A. Seragnoli, Department of Experimental, Diagnostic and Specialty Medicine - DIMES, University of Bologna, Italy; ^2^ Haematopathology Unit, Department of Experimental, Diagnostic and Specialty Medicine - DIMES, University of Bologna, Italy

**Keywords:** Diffuse Large B-cell Lymphoma, genomic instability, CHK1, CHK2, gamma-H2AX, MYC

## Abstract

The recent finding that *MYC*-driven cancers are sensitive to inhibition of the DNA damage response (DDR) pathway, prompted us to investigate the role of DDR pathway as therapeutic target in diffuse large B-cell lymphoma (DLBCL), which frequently overexpresses the *MYC* oncogene. In a preliminary immunohistochemical study conducted on 99 consecutive DLBCL patients, we found that about half of DLBCLs showed constitutive expression of the phosphorylated forms of checkpoint kinases (CHK) and CDC25c, markers of DDR activation, and of phosphorylated histone H2AX (γH2AX), marker of DNA damage and genomic instability. Constitutive γH2AX expression correlated with c-MYC levels and DDR activation, and defined a subset of tumors characterised by poor outcome. Next, we used the CHK inhibitor PF-0477736 as a tool to investigate whether the inhibition of the DDR pathway might represent a novel therapeutic approach in DLBCL. Submicromolar concentrations of PF-0477736 hindered proliferation in DLBCL cell lines with activated DDR pathway. These results were fully recapitulated with a different CHK inhibitor (AZD-7762). Inhibition of checkpoint kinases induced rapid DNA damage accumulation and apoptosis in DLBCL cell lines and primary cells. These data suggest that pharmacologic inhibition of DDR through targeting of CHK kinases may represent a novel therapeutic strategy in DLBCL.

## INTRODUCTION

Diffuse large B cell lymphoma (DLBCL), the most common non Hodgkin lymphoma (NHL) subtype, is characterized by an aggressive clinical course [1], and standard first line R-CHOP (Rituximab, Cyclophosphamide, Doxorubicin, Vincristine, Prednisone) chemoimmunotherapy results in approximately 60% cure rates [2].

In the last ten years gene expression profiling has identified two distinct signatures strongly related to the outcome and response to therapy, the germinal center (GCB) B-cell and the activated B-cell (ABC) signatures [3–5], but this classification did not translate yet in a tailored therapeutic approach, as the International prognostic index (IPI) risk score (based on clinical variables), remains the most reliable prognostic tool available in clinical practice [2]. Interestingly, recent data show that a significant fraction of DLBCL display chromosomal segregation defects and aneuploidy, a prominent feature of instable genomes, and these characteristics have been demonstrated to correlate with poor prognosis following conventional R-CHOP chemoimmunotherapy [6]. Accordingly, whole exome sequencing studies showed that DLBCLs are characterized by a particularly large number of genomic aberrations [7]. Moreover a consistent percentage of DLBCLs are characterized by increased c-MYC expression [8–10] which is linked mechanistically to instrinsic genomic instability by a mechanism called replicative stress, consisting in DNA damage accumulation during the S phase of the cell cycle [11].

Genomic instability is now considered a hallmark of cancer, which indicates a tendency toward accumulation of DNA damage with progressive acquisition of mutations and genomic abnormalities, favouring cancer development, metastatic phenotypes and chemoresistance [11, 12]. Following DNA damage, cells normally respond by activating the DNA damage repair pathway (DDR), which results in cell cycle arrest, DNA repair or eventually p53-mediated cell death [13]. The ataxia telangiectasia mutated (ATM) and ataxia telangiectasia and RAD3-related protein (ATR) kinases are activated following DNA double and/or single strand breaks and in turn they phosphorylate the downstream targets checkpoint kinases (CHK) 1 and 2, and the histone H2AX, which is considered to be a reliable DNA damage marker [13–15]. CHK1 and CHK2 activation triggers the inhibitory phosphorylation of the CDC25 phosphatases, which results in delayed mitotic entry, ensuring appropriate DNA repair at the G2/M checkpoint [13].

Accordingly it has been recently demonstrated that tumours bearing high levels of oncogene-induced replicative stress, such as *MYC* driven aggressive lymphoma mouse models and neuroblastoma, are sensitive to single agent CHK inhibitors [16–18].

On the other hand recent studies show that a subset of DLBCLs display mutations of genes involved in DNA repair [19]. Although the functional consequences of specific mutations have not been elucidated yet, these data further highlight the role of the DDR pathway in DLBCL pathogenesis. Therefore, inhibition of the DNA damage repair pathway may represent a valid therapeutic approach to fight cancers with aberrant DDR activation and CHK inhibitors are currently being tested in clinical trials in combination with DNA damaging agents (chemotherapy and radiotherapy) in a variety of tumors [20,21]. Taken together these findings represent a strong rationale to investigate the functional role of the DDR pathway in DLBCL, and to ascertain whether its components might represent potential therapeutic targets. Here we demonstrated that 1) a substantial fraction of DLBCLs display constitutive expression of the DNA damage marker γH2AX, which was associated with poor prognosis following conventional R-CHOP/CHOP-like chemoimmunotherapy, 2) that c-MYC expression, γH2AX and DDR activation were significantly associated, confirming the intimate relationship between oncogene–induced genomic instability and DDR activation in DLBCL, and 3) that DLBCL cell lines and primary cells exhibiting constitutive activation of the DDR pathway are very sensitive to the inhibition of checkpoint kinases. Taken together these data suggest that pharmacologic inhibition of DDR through targeting of CHK kinases may represent a new promising therapeutic strategy in the subset of DLBCLs with activated DDR pathway.

## RESULTS

### Constitutive activation of DDR components and genomic instability in diffuse large B-cell lymphomas

We assessed by immunohistochemistry the expression levels of the components of the DDR pathway (CHK1, CHK2, CDC25c) and their phosphorylated forms in three reactive lymphnodes, 27 cases of small lymphocyte lymphoma (SLL), 18 marginal zone lymphoma (MZL), 44 Hodgkin lymphoma (HL), 22 Burkitt lymphoma (BL), and 99 consecutive DLBCL cases diagnosed at our Institution from 2002 to 2011.

Components of the DDR pathway CHK1, CHK2 and CDC25c resulted to be expressed in 100% of B cell neoplasms and normal reactive follicles tested (Table 1) but only aggressive lymphomas (BLs and DLBCLs) showed a significant activation of DDR pathway, as demonstrated by the expression of CHK1, phosphorylated at ser 345, and CDC25c, phosphorylated at ser 216 (Table 1). The phosphorylated form of the CHK2 kinase at thr 68 was found to be expressed only in a minority of DLBCL cases (5%) (Table 1).

**Table 1:** Immunohistochemical results

Tumor type	CHK1		pCHK1		CHK2		pCHK2		CDC25		pCDC25		γH2AX	
	pos	(%)	pos	(%)	pos	(%)	pos	(%)	pos	(%)	pos	(%)	pos	(%)
**RL**	3/3	(100)	0/3	(0)	3/3	(100)	0/3	(0)	3/3	(100)	0/3	(0)	0/3	(0)
**CLL/SLL**	27/27	(100)	2/27	(7)	27/27	(100)	0/27	(0)	27/27	(100)	0/27	(0)	3/27	(11)
**SMZL**	18/18	(100)	2/18	(11)	18/18	(100)	0/18	(0)	18/18	(100)	0/18	(0)	1/18	(5)
**CHL**	44/44	(100)	6/44	(14)	44/44	(100)	3/44	(7)	44/44	(100)	9/44	(20)	8/44	(18)
**BL**	22/22	(100)	10/22	(45)	22/22	(100)	3/22	(14)	22/22	(100)	18/22	(82)	5/22	(23)
**DLBCL**	99/99	(100)	38/99	(38)	99/99	(100)	5/99	(5)	99/99	(100)	40/99	(40)	47/99	(47)

Abbreviations: positive (pos), negative (neg), reactive lymph nodes (RL), chronic lymphocytic leukaemia/small lymphocyte lymphoma (SLL), marginal zone lymphomas (MZL), classical Hodgkin lymphoma (CHL), Burkitt lymphoma (BL), diffuse large B –cell lymphomas (DLBCL).

**Figure 1:**
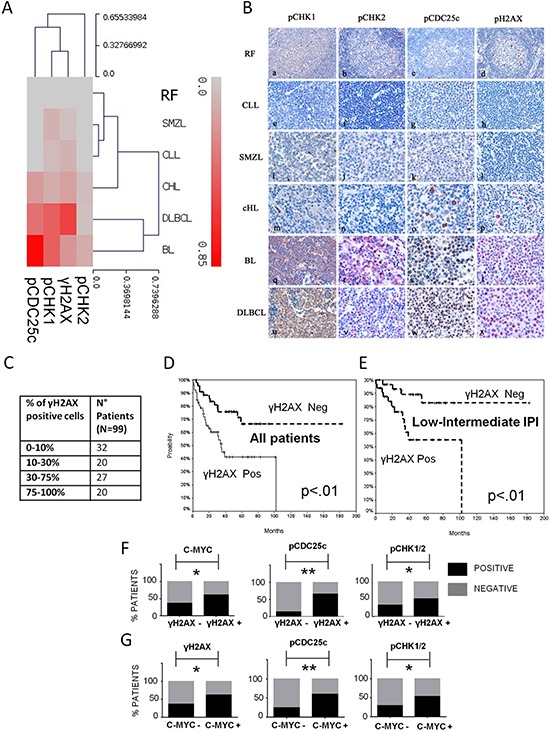
The DDR pathway is aberrantly active in DLBCL **(A)** Hierarchical clustering, displayed in a dendrogram, showing higher constitutive activation of the DDR pathway and γH2AX staining in aggressive non Hodgkin B-cell lymphomas (DLBCL and BL), compared to indolent B-cell lymphoma subtypes (CLL, SMZL), HL and reactive follicles (RF). The level of expression in each lymphoma type and in RF, that is the number of positive cases/number of evaluable cases, is represented by a color scale, starting from grey, indicating 0% of the samples positive, up to brilliant red, indicating 85% of the samples positive (the maximum percentage of positivity detected was 82%). The highest levels of γH2AX and pCHK1ser 345 staining were observed in DLBCL, with 47% and 38% of cases expressing respectively γH2AX and pCHK1 ser 345 in more than 30% of cells. **(B)** Representative TMA spots of reactive follicles (RF) (a-d), CLL (e-h), SMZL (i-l) HL (m-p), BL (q-t) and DLBCL (u-x) stained for pCHK1, pCHK2, pCDC25c and γH2AX. Higher expression levels of activated DDR pathway components and γH2AX were observed in DLBCL and BL, compared to indolent NHL subtypes and HL, showing low or absent DDR activation and γH2AX staining. **(C)** Table showing the spectrum of γH2AX expression in our DLBCL cohort. **(D)** Overall survival (OS) curve of γH2AX positive compared to γH2AX negative DLBCL patients. 99 consecutive DLBCL patients diagnosed and treated at our institution with Rituximab plus CHOP/CHOP-like regimens from 2002 to 2011 were considered. Patients positive (pos) for γH2AX at initial diagnosis had a significant inferior OS rates compared to γH2AX negative (neg) ones (with 5-year OS rate of 41% vs 70% respectively, *p* < 0.01, log-rank test). **(E)** Overall survival curve of the low-intermediate risk IPI group according to the γH2AX status. 63 patients with low-intermediate risk IPI score (0–2) were considered in this subgroup analysis. The 5-year OS of γH2AX positive patients resulted significantly lower compared to the OS of γH2AX negative patients, with 5-year OS rate of 55% vs 83% respectively (*p* < 0.01, log-rank test). **(F)** Bar graphs showing a significantly increased incidence of c-MYC, pCDC25c, and pCHK1/2 positive cases in the γH2AX positive subgroup, compared to the γH2AX negative subgroup. The incidence of c-MYC positive cases raised from 35% to 62%, from the γH2AX negative to γH2AX positive group (*p* = 0.02) (Fisher's exact test). *P < 0.05; **P < 0.005. The incidence of pCDC25c positive cases increased from 17% to 66% from the γH2AX negative to the γH2AX positive group (*p* < 0.001) (Fisher's exact test). The incidence of pCHK1/2 positive cases increased from 33% to 51% from the γH2AX negative to the γH2AX positive group (p=0.04) (Fisher's exact test). **(G)** Bar graphs showing a significantly increased incidence of γH2AX, pCDC25c, and pCHK1/2 positive cases in the c-MYC positive subgroup, compared to the c-MYC negative subgroup. The 3 cases with missing c-MYC values were excluded from this analysis.

We thus hypothesized that lymphomas with constitutive activation of the DDR pathway would be characterized by higher levels of inherent genomic instability. In order to verify this hypothesis we investigated the expression of the phosphorylated form of the histone H2AX at serine 139 (γH2AX), a marker of DDR activation and DNA double strand breaks [13–15], in our B-cell lymphoma panel. Remarkably DLBCLs showed the highest constitutive γH2AX expression with 47% of positive cases (defined as percentage of positive cells ≥ 30%, in the methods section), confirming that DLBCL is a neoplasm characterized by high genomic instability and inherent DNA damage (Figure 1A, 1B). Reactive follicles and indolent B-cell lymphomas (MZL and CLL) showed low or absent expression of activated DDR components and γH2AX, and Hodgkin lymphoma cases showed intermediate expression (18% of γH2AX positive cases) (Table 1, Figure 1A, 1B).

By using cluster analysis on immunohistochemical results, considering the whole panel of DDR activation markers, aggressive B-cell neoplasms (DLBCL and BL) clearly clustered together, being characterized by higher constitutive CHK1, CDC25c, and H2AX phosphorylation, whereas indolent B-cell neoplasms and HL formed a separate cluster (Figure 1A).

Since high inherent genomic instability favours cancer progression and chemoresistance we next investigated the prognostic significance of constitutive γH2AX expression and DDR activation in DLBCL patients. All patients were diagnosed and treated with chemoimmunotherapy at our institution. Characteristics of patients and univariate analyses are shown in Table S1. The spectrum of γH2AX expression is shown in Figure 1C. In the univariate analysis, pCDC25c ser 216 and γH2AX overexpression were significantly associated with worse overall survival (Table S1), as well as age≥60 years, IPI score > 2 and bcl-2/MYC double positivity. Remarkably 5-year OS was 41% for γH2AX positive vs 70% for γH2AX negative patients (Figure 1D). Interestingly, the prognostic significance of γH2AX was particularly evident in the low-intermediate risk IPI group (0–2 risk factors), identifying a subgroup characterized by worse outcome (55% 5-year OS) (Figure 1E). In the multivariate framework γH2AX was found to be an independent prognostic predictor (Table S2). These results suggest that constitutive γH2AX expression is an independent negative prognostic predictor in DLBCL, being able to identify patients with poor prognosis even within the low-intermediate risk group according to the IPI score.

### c-MYC, γH2AX and DDR activation markers are frequently co-expressed in DLBCL

Since it has been reported that *MYC*-driven replicative stress leads to inherent DNA damage [11, 12, 17, 18], we evaluated the correlation between c-MYC, γH2AX (marker of DNA damage) and pCHK1/2 (pCHK1+pCHK2) and pCDC25c expression (markers of DDR activation), hypothesizing that γH2AX positive DLBCLs would be characterized by c-MYC overexpression and enhanced DDR activation.

The 99 patients considered in the survival analysis were divided in 2 groups based on γH2AX expression levels. By using Fisher's exact test we found a statistically significant association between γH2AX positivity, c-MYC expression, and increased activation of CDC25c and CHK kinases (Figure 1F) (Table 2). Interestingly non-GCB and GCB cases, low and high risk IPI score patients were equally distributed in the γH2AX positive and negative groups (Table 2).

**Table 2:** Characteristics of γH2AX negative and positive DLBCL patients with respect to IPI score, p53 expression levels, cell of origin (GCB vs non-GCB), Bcl-2 expression, c-MYC expression, and DDR pathway activation (pCHK1/2, pCDC25c)

Factor	γH2AX− (N=52)	γH2AX+ (N=47)	Missing	*p*-value (Fisher's exact test)
**IPI**			0	0.67
Low (0–2)	34 (65%)	29 (62%)		
High (3–4)	18 (35%)	18 (38%)		
**p53**			2 (2%)	0.16
< 50%	42 (81%)	32 (68%)		
≥ 50%	9 (17%)	14 (30%)		
**Ki-67**			0	0.82
< 90%	35 (67%)	31 (66%)		
≥ 90%	17 (33%)	16 (34%)		
**Cell of Origin**			0	0.45
GCB	26 (50%)	20 (43%)		
Non-GCB	26 (50%)	27 (57%)		
**Bcl-2**			0	0.89
< 70%	15 (29%)	13 (28%)		
≥ 70%	37 (71%)	34 (72%)		
**c-MYC**			3 (3%)	**0.01**
< 40%	31 (60%)	18 (38%)		
≥ 40%	18 (35%)	29 (62%)		
**pCHK1/2**			0	**0.04**
< 30%	35 (67%)	23 (49%)		
≥ 30%	17 (33%)	24 (51%)		
**pCDC25c**			**0**	< **0.001**
< 30%	43 (83%)	16 (34%)		
≥ 30%	9 (17%)	31 (66%)		

*P* value was calculated with the Fisher's exact test

Also by categorizing patients in 2 groups depending on the c-MYC expression status, we could confirm the association between c-MYC, γH2AX, pCDC25c and pCHK1/2 kinases expression: of note γH2AX positive cases raised from 37% to 63%, pCHK1/2 positive cases from 28% to 55%, pCDC25c positive cases from 24% to 55% from the c-MYC negative to the c-MYC positive group (Figure 1G). These results indicate a tight association between c-MYC expression, inherent genomic instability, and constitutive DDR activation.

### Inhibition of checkpoint kinases hinders proliferation and promotes apoptotic death in DLBCL cells

The observation that about half of the DLBCL cases were characterised by constitutive γH2AX expression, which was associated with adverse outcome and with activation of the DDR pathway, suggests that inhibition of the DDR may represent a promising new therapeutic strategy to fight this subset of tumours.

Therefore, we explored the effect of DDR pathway inhibition in a series of human B-cell NHL cell lines, including 6 DLBCL cell lines, 1 BL and 1 HL cell line (used respectively as a positive and negative control for constitutive DDR activation). All the DLBCL cell lines, irrespectively of their GC or ABC origin, and the BL cell line RAMOS showed some activation of the DDR pathway, as defined by constitutive phosphorylation of CHK1 at ser 345 and/or phosphorylation of CHK2 at thr 68, and constitutive expression of γH2AX, evaluated by Western blot analysis.

**Figure 2:**
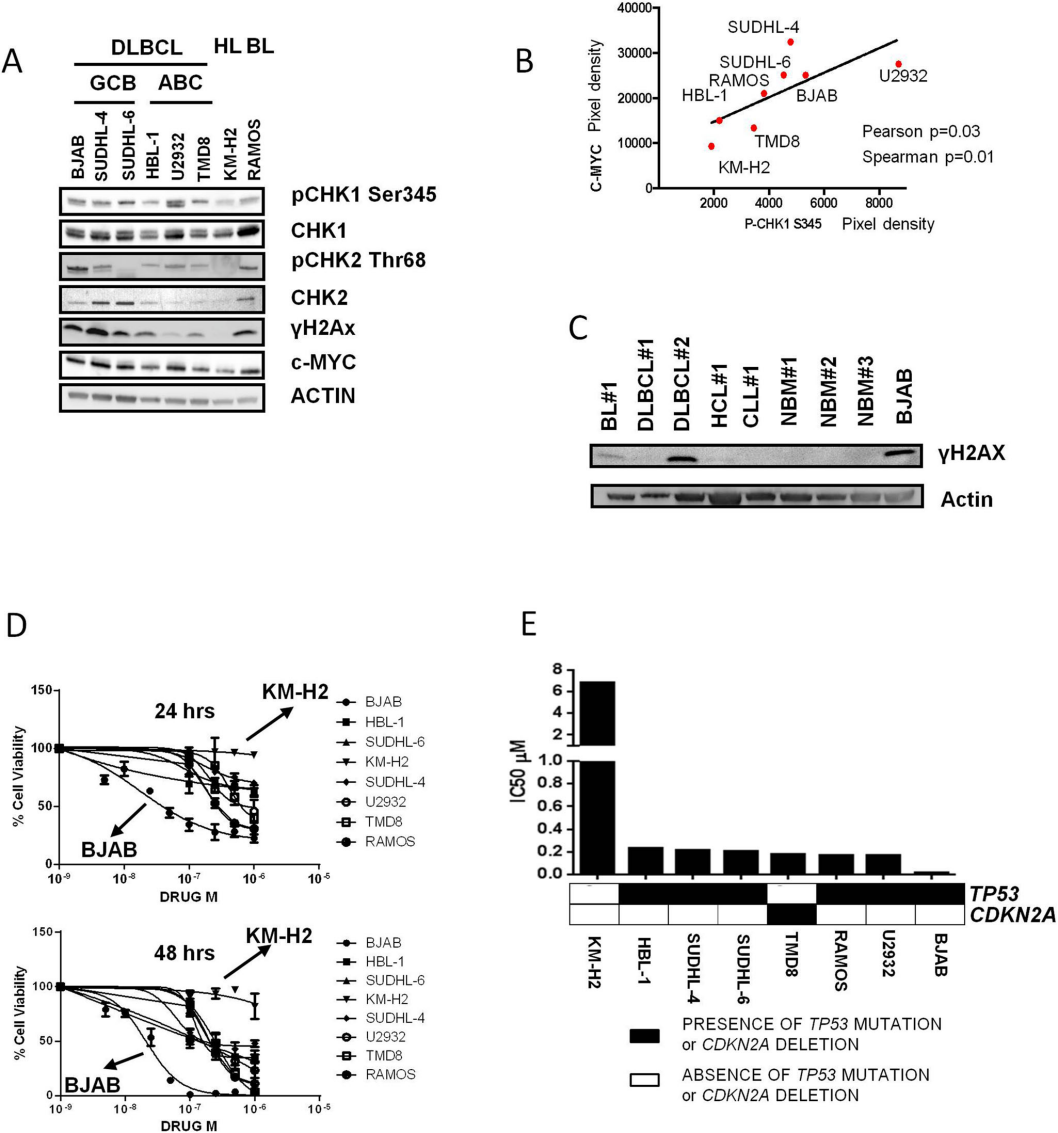
The CHK inhibitor PF-0477736 shows potent antiproliferative activity in aggressive B-cell lymphoma cells **(A)** Western immunoblotting showing the activation status of the DDR pathway and the expression of the DNA damage marker γH2AX in DLBCL, BL, and HL cells. The GCB-derived DLBCL cell lines BJAB, SUDHL-4, SUDHL-6, the ABC-derived cell lines HBL-1, U-2932, TMD8, the BL cell line RAMOS and the HL cell line KM-H2 were harvested during the exponential growth phase, and assessed for the expression levels of pCHK1 ser345, CHK1, pCHK2 thr 68, CHK2, γH2AX, and c-MYC. **(B)** Graph showing correlation between c-MYC and pCHK1 ser 345 expression levels in our panel of B-cell lymphoma cell lines, as evaluated by densitometry analysis using the ImageJ software (National Institutes of Health, Bethesda, MD). Pixel density is expressed in arbitrary units. The *p* value of the correlation was calculated with Spearman and Pearson's tests. **(C)** Western blot assay showing the expression of the DNA damage marker γH2AX in primary aggressive B-cell lymphoma cells, indolent B-cell lymphoma cells, and normal bone marrow progenitors. BL (Burkitt lymphoma), DLBCL (Diffuse large B-cell lymphoma), CLL (Chronic Lymphocytic Leukemia), HCL (Hairy Cell Leukemia), NBM (Normal bone marrow mononucleated cells). The BJAB cell line was used as a positive control. **(D)** WST-1 assay of DLBCL, BL, HL cell lines treated with increasing concentrations of the CHK 1/2 kinase inhibitor PF-0477736 for 24 and 48 hours. Each value is the mean of three independent experiments performed in triplicate. Error bars represent standard error of the mean (s.e.m). **(E)** IC50 values after 48 hours of incubation with PF-0477736. After 48 hours of incubation with PF-0477736 the DLBCL GCB-derived BJAB cells were the most sensitive with an IC50 value of 9 nM. All other DLBCL cell lines were sensitive to nanomolar concentrations of PF-0477736, displaying IC50 values between 160 and 230 nM. Only the HL derived KM-H2 cells were resistant to treatment with an IC50 value of 7 μM at 48 hours. The black and white squares represent the presence or absence of *TP53* mutations and/or *CDKN2A* deletions in DLBCL cells.

The HL cell line KMH2 showed low or absent basal levels of activation of the DDR pathway, which did not translate in increased γH2AX expression (Figure 2A). In line with the results of the immunohistochemical study, we found a significant correlation between c-MYC levels and pCHK1 ser 345 levels (Figure 2B). Finally, primary cells from 2 out of 3 aggressive B-cell lymphoma patients (2 DLBCL, 1 BL) showed constitutive γH2AX expression. We did not find γH2AX overexpression in indolent B-cell lymphoma primary cells and normal bone marrow mononucleated cells (Figure 2C).

To evaluate the therapeutic potential of CHK inhibition, we incubated B-cell lymphoma cell lines with increasing concentrations of PF-0477736 from 5 nM to 10 μM. Cell viability was assessed by WST-1 assay (Roche). PF-0477736 is a selective ATP competitive CHK inhibitor with IC50 of 2 and 47 nM respectively for CHK1 and CHK2, which was demonstrated to have activity in preclinical models of solid tumours in combination with DNA damaging agents [22, 23]. PF-0477736 induced cell death in a time and dose dependent manner, with a maximal effect after 48 hours. The most sensitive cell line was BJAB, with an IC50 of 48 nM and 9nM at 24 and 48 hours (hrs) respectively (Figure 2D, 2E). KM-H2 cells were resistant. All the other DLBCL cell lines were sensitive to nanomolar concentrations of PF-0477736, with IC50s ranging from 160 to 230 nM (Figure 2D, 2E). The resistant KM-H2 cells did not display any constitutive expression of γH2AX and pCHK2, and only weak pCHK1 and c-MYC expression (Figure 2A). These results were confirmed also by using a different inhibitor of CHK kinases, AZD-7762 [24], which also displayed submicromolar IC50 values at 48 hours (Fig S1) in DLBCL and BL cell lines. Taken together these observations indicate a high efficacy of CHK inhibitors against DLBCL cell lines characterized by constitutive activation of the DDR pathway.

Since some reports link the sensitivity to CHK inhibition to the dysfunction of the p53 axis in different tumor models [22, 24], we profiled our cell lines by conventional Sanger sequencing for the presence of *TP53* mutations and *CDKN2A* deletions, (known recurrent genomic alterations in DLBCL which result in p53 axis and G1/S checkpoint dysfunction). Alterations resulting in G1/S checkpoint dysfunction were found in all the DLBCL cell lines and in the BL cell line RAMOS and only the resistant KM-H2 cells were *TP53* wild type with no alteration of *CDKN2A* (Figure 2E) (Table S3). Notably, in line with previous reports [22, 24], co-treatment with the CHK inhibitor PF-0477736 enhanced the efficacy of doxorubicin in the *TP53* mutant DLBCL cell lines (Figure S2A).

In order to establish the relevance of p53 status in the cytotoxic activity of PF-0477736, we induced stable transfection of the *TP53/* wild-type KM-H2 cells with the dominant negative mutant p53dd. Interestingly we failed to induce sensitivity to PF-0477736 single agent or in combination with doxorubicin after p53dd transfection (Figure S2 B–D). These results indicate that at least in this cellular context disruption of p53 function is not sufficient to induce sensitivity to CHK inhibition or to combinations with doxorubicin, and that probably other mechanisms cooperate in determining the sensitivity to CHK inhibition and dependence on the G2/M checkpoint in these cells.

**Figure 3:**
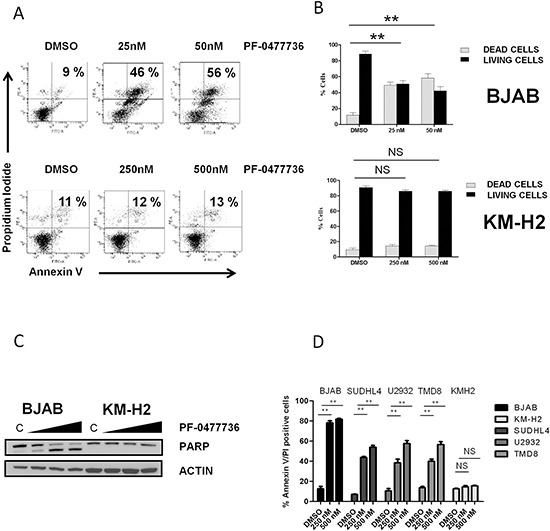
PF-0477736 induces cell death by apoptosis in DLBCL cell lines **(A)** Representative experiment demonstrating the effect of different doses of PF-0477736 (25 or 50nM for 24 h in BJAB cells, 250 and 500 nM for 24 h in KM-H2 cells) on apoptosis as determined by annexin V-propidium iodide (PI) binding assay. The percentage of dead cells is shown in the upper right quadrant. **(B)** Summary of the results of dual annexin V and propidium iodide (PI) staining. Each value is the mean of three independent experiments performed in triplicate. Error bars represent s.e.m. *P < 0.05; **P < 0.005; NS (not significant). **(C)** Immunoblotting showing cleavage of poly (adenosine diphosphate ribose) polymerase (PARP) in BJAB cells after 24 hours of incubation with increasing doses (50, 250, 500 nM) of PF-0477736, with respect to control (C) (DMSO). Consistent the results of annexin V and propidium iodide staining no PARP cleavage was observed in the resistant KM-H2 cells following treatment. **(D)** Summary of the results of dual annexin V – (PI) staining for different DLBCL cell lines. Each value is the mean of three independent experiments performed in triplicate. Error bars represent s.e.m. *P < 0.05; **P < 0.005.

To further investigate the mechanisms of action of PF-0477736 we determined the effect on apoptosis by using Annexin V/Propidium Iodide staining. In line with WST-1 assay results, low nanomolar concentrations of PF-0477736 induced cell death by apoptosis after 24 hours of incubation in the sensitive BJAB cells, but not in the resistant KM-H2 cells (Figure 3A, 3B). Consistent with the induction of apoptosis PARP cleavage was detected by western blot after 24 hours in BJAB cells (Figure 3C).

Nanomolar concentrations of PF-0477736 induced apoptotic cell death also in the other DLBCL cell lines tested at 48 hours (Figure 3D).

### PF-0477736 inhibits CDC25c phosphorylation and determines rapid DNA damage accumulation in DLBCL cell lines

Following DNA damage, the activation of CHK1 and CHK2 results in the inhibitory phosphorylation of the CDC25c phosphatase, which determines inhibition of mitotic entry, allowing DNA repair [13]. Therefore, the impairment of the G2/M checkpoint mediated by CHK inhibition should determine an uncontrolled DNA damage accumulation leading to replication fork collapse followed by cell death.

**Figure 4:**
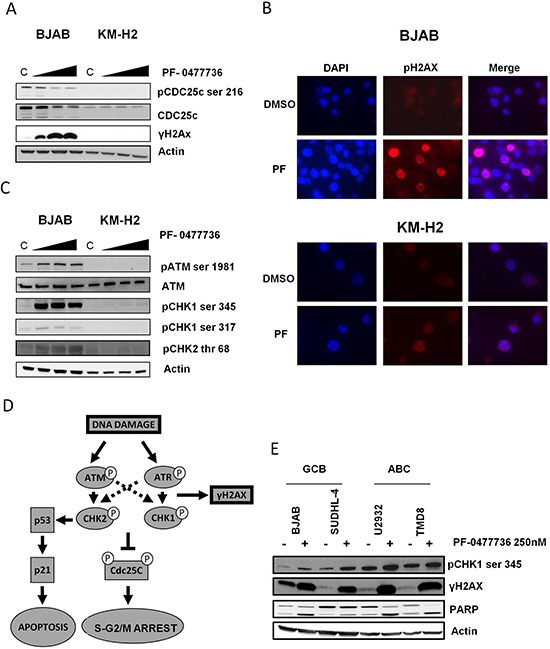
PF-0477736 inhibits CDC25c phosphorylation and determines DNA damage accumulation in DLBCL cells **(A)** Western blot assay of BJAB and KM-H2 cells treated with increasing concentrations of PF-0477736 (50, 250, 500 nM) for 24 hours, showing inhibition of the donwstream target pCDC25c ser 216 and contemporary upregulation of the DNA damage marker γH2AX in the sensitive BJAB cells. On the contrary we could not detect any γH2AX induction in the resistant KM-H2 cells. Of note KM-H2 cells did not show any basal CDC25c phosphorylation, consistent with the low level of DDR activation shown in Figure 1. C (control, DMSO). **(B)** Early effects of PF-0477736 on γH2AX phosphorylation levels. Immunofluorescence of BIAB and KM-H2 cells indicating increased γH2AX levels as soon as after 3 hours of incubation with PF-0477736 (PF) 250 nM in the BJAB cells. Consistent with Fig. 5A, the resistant KM-H2 cells did not show significant γH2AX upregulation following treatment. **(C)** Effects of PF-0477736 on the upstream components of the DDR pathway. BJAB and KM-H2 cells were incubated with increasing concentrations of PF-0477736 (50, 250, 500 nM) for 24 hours and effects on ATM, CHK1 and CHK2 phosphorylation assessed by western blot. Consistent with DNA damage induction, PF-0477736 triggered a futile feedback loop in the sensitive BJAB cells, with increased ATM activation resulting in hyperphosphorylation of CHK1 and CHK2. C (control, DMSO). **(D)** Model of activation of the DDR pathway and G2/M checkpoint after DNA damage. After DNA damage, the histone H2AX is promptly recruited at the DNA damage foci and phosphorylated. The ATR and ATM kinases phosphorylate CHK1 and CHK2 which in turn phosphorylate the downstream target CDC25c phosphatase. This inhibitory phosphorylation results in cell cycle arrest, allowing DNA repair. The CHK2 kinase participates also in the G1/S checkpoint, by stabilizing p53 after DNA damage. **(E)** The GCB-derived SUDHL-4 cells and the ABC-derived U-2932 and TMD8 cells were incubated with PF-0477736 250 nM for 24 hours. BJAB cells were used as a control. Consistent with the data shown in Fig. 5A and 5B, an induction of γH2AX and pCHK1 ser 345 was observed in all DLBCL cell lines. At the same time point, PARP cleavage consistent with apoptosis induction was detected in all DLBCL cell lines.

To verify this hypothesis we first incubated the sensitive BJAB cells and the resistant KM-H2 cells with increasing concentrations of PF-0477736 and assessed effects on CDC25c and γH2AX phosphorylation. PF-0477736 potently inhibited the phosphorylation of CDC25c at ser 216 in the sensitive BJAB cells. As CDC25c de-phosphorylation triggers its proteasome dependent degradation, in line with recent findings [25] we also observed a decrease in total CDC25c levels following treatment. Moreover we observed a rapid and marked dose dependent increase of γH2AX consistent with accumulation of DNA damage (Figure 4 A, 4B). On the contrary, in KM-H2 cells we did not observe increased γH2AX levels following PF-0477736 treatment (western blot and immunofluorescence), and consistent with the low activation status of the DDR pathway, these cells did not show any constitutive CDC25c phosphorylation (Figure 4A). Taken together, these findings show that inhibition of the CHK kinases effectively increases the amount of DNA damage in DLBCL cells without the presence of exogenous genotoxic sources. In line with these results, incubation with PF-0477736 for 24 hrs triggered a futile feedback loop resulting in increased phosphorylation of ATM, and both its targets CHK1 and CHK2, as previously reported with different CHK inhibitors [26] (Figure 4C). The position and function of different components of the DDR pathway is briefly elucidated in Figure 4D.

A similar mechanism resulting in DNA damage accumulation and hyper-phosphorylation of CHK1, was observed in all the DLBCL cell lines tested (Figure 4E).

### PF-0477736 shows high single agent activity and promotes DNA damage in primary aggressive B-cell lymphoma cells

To investigate the activity of PF-0477736 in primary lymphoma cells and to evaluate its toxicity on bone marrow mononucleated cells we incubated a panel of primary cells from aggressive B-cell lymphoma patients (n = 5) [2 BL, 2 DLBCL, 1 mantle cell lymphoma blastoid variant (MCL)], indolent B-cell lymphoma patients (n = 5) (3 CLL, 1 MZL, 1 hairy cell leukemia) and normal bone marrow progenitors (n = 7), with PF-0477736 at the fixed dose of 500 nM for 24 hours.

**Figure 5:**
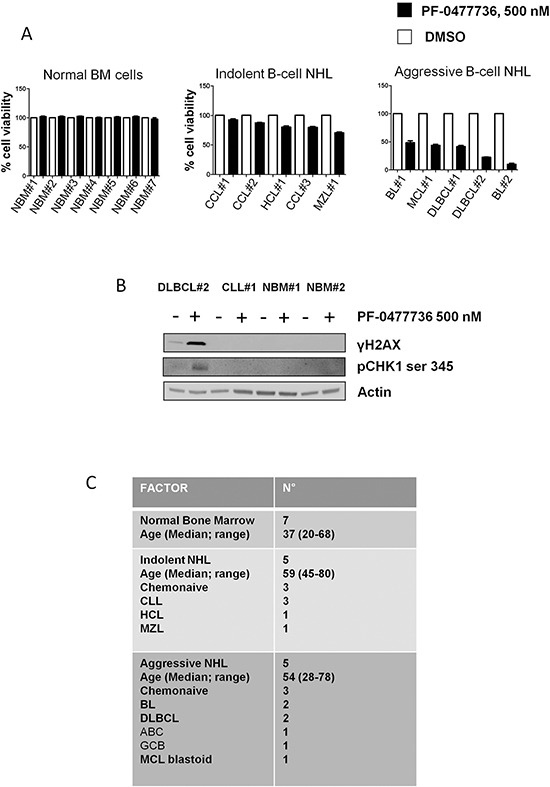
Effects of PF-0477736 in primary lymphoma cells and bone marrow mononucleated cells **(A)** Normal bone marrow mononucleated cells harvested from 7 patients with no signs of bone marrow involvement, at the moment of initial diagnosis, 5 primary indolent B-cell lymphoma samples, and 5 primary aggressive B-cell lymphoma samples were incubated with PF-0477736 500 nM for 24 hours, and cell viability evaluated by trypan-blue staining. The viability of each primary sample was normalized to its own untreated control. Error bars represent s.e.m. BL (Burkitt lymphoma), DLBCL (Diffuse large B-cell lymphoma), CLL (Chronic Lymphocytic Leukemia), HCL (Hairy Cell Leukemia), NBM (Normal bone marrow mononucleated cells), MZL (Marginal zone lymphoma), MCL (Mantle cell lymphoma). **(B)** Western blot assay showing on treatment modifications of γH2AX and pCHK1 ser 345 in primary cells. Similarly to what we observed in cell lines, treatment with PF-0477736 determined DNA damage accumulation with increased γH2AX and pCHK1 ser 345 levels in DLBCL primary cells, whereas no changes were observed in primary B-CLL cells and normal bone marrow mononucleated cells (NBM). **(C)** Table showing characteristics of patients whose primary cells were harvested and used for the experiments above.

Consistently with our previous findings, aggressive B-cell lymphoma primary cells were sensitive to PF-0477736, whereas primary indolent B-cell lymphoma cells were only slightly sensitive and normal bone marrow progenitors were resistant (Figure 5A). Of note, in DLBCL primary cells PF-0477736 induced DNA damage accumulation with increase of γH2AX and pCHK1, as already demonstrated in DLBCL cell lines (Figure 5B). Characteristics of patients whose cells were used for *ex-vivo* experiments are shown in Figure 5C.

PF-0477736 displayed activity both in *TP53* wild type and mutant primary cells, although the highest activity was observed in a *TP53* mutant Burkitt lymphoma sample (BL#2) (Figure 5B) (Table S3).

These data strongly suggest that PF-0477736 may exert a selective cytotoxic activity on aggressive lymphoma cells, without inducing specific changes in normal bone marrow mononucleated cells, at least in short term culture.

## DISCUSSION

In this study we addressed the value of targeting the DDR pathway in aggressive B-cell lymphomas, focusing the attention on DLBCL. First we demonstrated that DLBCL is a neoplasm characterized by high inherent genomic instability and that the DDR pathway is aberrantly active in DLBCL cell lines, primary cells, and DLBCL tissue samples. In our patient cohort about half of all patients showed high levels of the DNA damage marker γH2AX and activation of the DDR components at the moment of initial diagnosis. We observed a significant correlation between γH2AX expression levels, constitutive DDR activation and c-MYC overexpression, suggesting an intimate relationship between *MYC*-induced oncogenic stress, genomic instability and DDR activation in DLBCL (Fig. 1E). Moreover, patients showing high γH2AX expression had a statistically significant lower 5-year OS compared to patients with low levels of γH2AX following conventional chemoimmunotherapy (Fig. 1C, 1D). These findings indicate that constitutive DDR activation could determine resistance to therapy with DNA damaging agents and that the DDR could be an important therapeutic target in DLBCL.

We demonstrated in *in vitro* and *ex-vivo* models that targeting the DDR pathway by inhibition of CHK kinases could be an effective treatment strategy in the subset of DLBCL with constitutive activation of DDR. Treatment with the CHK1/2 inhibitor PF-0477736 as single agent induced cell death by apoptosis at nanomolar concentrations in DDR positive DLBCL cell lines and markedly inhibited cell viability in primary DLBCL and BL cells. The antiproliferative affects observed with PF-0477736 in DLBCL cells were recapitulated by using a different inhibitor of CHK kinases (AZD7762), indicating a specific class effect of these compounds in DLBCL. Inhibition of CHK kinases effectively resulted in inhibition of the phosphorylation of the downstream target CDC25c, and increased the phosphorylation of the DNA damage marker γH2AX both in DLBCL cell lines and primary cells. Interestingly the increased amount of DNA damage determined by CHK inhibition triggered a futile feed-back loop characterized by enhanced ATM activation and hyperphosphorylation of CHK1 and CHK2, which could be used as a biomarker of activity in future clinical trials. Notably we did not observe any *in vitro* cytotoxicity in normal bone marrow progenitors. Our results also indicate that the sensitivity to CHK inhibition of the DDR negative *TP53*-wild type KM-H2 cells was unaffected by transfection with the dominant negative p53 mutant p53DD, suggesting that the p53 status is not a primary determinant of sensitivity to DDR inhibition, and that the presence constitutive DDR activation likely plays a major role. On the other hand it is noteworthy that single agent PF-0477736 showed activity in *TP53* mutant aggressive B-cell lymphoma cell lines and primary cells, and that combined treatment with PF-0477736 was able to revert resistance to doxorubicin in *TP53 mutant cells* (Figure S2). These observations suggest that inhibition of checkpoint kinases could be an effective treatment strategy for *TP53* mutant chemoresistant lymphomas with aberrantly active DDR pathway. These data are in line with previously published work showing single agent activity of CHK inhibitors in *MYC* driven lymphoma mouse models which recapitulate human Burkitt lymphoma [16], suggesting that Burkitt lymphoma and DLBCL share dysregulation of the DDR as a common pathogenetic feature. The role of DDR pathway and its upstream components [such as DNA dependent protein kinase (DNA-PK) and ATM] as therapeutic targets in *MYC* driven lymphomas has been confirmed in a recent report by Johnstone and coworkers on the efficacy of combined inhibition of ATM, DNA-PK and mammalian target of rapamycin (mTOR) in Eμ-MYC lymphoma models [27]. Intriguingly previous work from Blagosklonny and coworkers showed that enhanced DDR signaling was associated with mTOR over-activation during senescence coupled with hyper-mitogenic drive, and that inhibition of mTOR signaling by rapamycin attenuated DDR activation related to senescence [28, 29]; since oncogene-induced replicative stress may lead to cellular senescence and DDR activation [30], and given that active mTOR signaling and c-MYC expression have been demonstrated to be significantly associated in DLBCL [31], it is tempting to think that mTOR and *MYC* may cooperate in inducing aberrant DDR activation, and that dual targeting of DDR and mTOR could be a valuable strategy in DLBCL.

Moreover it has been recently reported that inherent DNA damage is a common trait shared by a variety of haematological cancer cell lines of both myeloid and lymphoid origin [32]. These findings, together with our observations, suggest that genomic instability, inherent DNA damage and alterations in DNA repair pathways may represent an Achilles heel by which highly genetically unstable aggressive lymphoid neoplasms may be targeted therapeutically.

**Figure 6:**
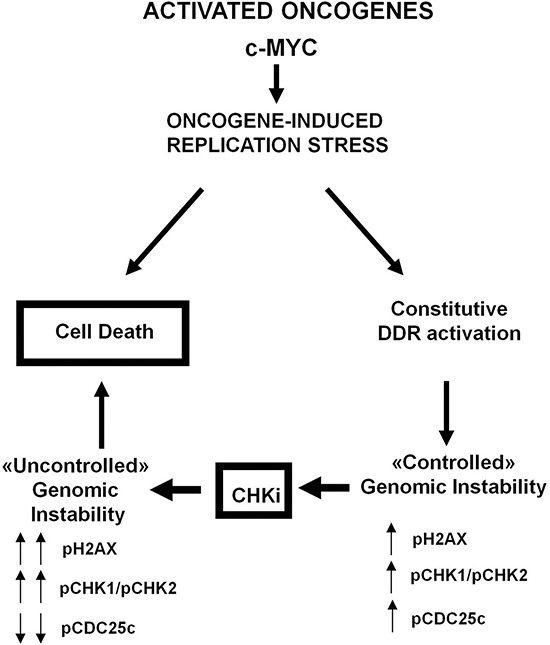
Proposed model of oncogene induced genomic instability in DLBCL Constitutive expression of oncogenes such as *MYC* increases replicative stress and determines G1/S checkpoint activation, which in normal cells leads to apoptotic cell death. Aggressive B-cell lymphoma cells tolerate increased amounts of genomic instability by constitutive activation of the G2/M checkpoint/DDR pathway (ATM-ATR-CHK1-CHK2-CDC25c axis). Inhibition of the DDR pathway by CHK inhibitors (CHKi) acts as a synthetic lethal mechanism, leading to “uncontrolled” genomic instability and cell death.

Collectively, our observations depict a model of synthetic lethality in which cells with high oncogene induced genomic instability carry higher levels of constitutive DDR activation in order to cope with oncogene-induced replicative stress, and are thus sensitive to CHK inhibition, according to the oncogene-induced DNA damage model for cancer development proposed by Halazonetis and coworkers [11] (Figure 6). In conclusion, our data demonstrate that inherent genomic instability and DDR pathway constitutive activation are frequent features in DLBCL, and are associated with a poor prognosis. The high efficacy of CHK inhibitors in DLBCL cell lines and primary cells characterised by aberrantly active DDR pathway, strongly suggest that the constitutive activation of this pathway may represent a novel therapeutic target, providing a rationale for further clinical evaluation of this therapeutic strategy in DLBCL.

## METHODS

### Immunohistochemistry and primary tissues

Ten tissue microarrays (TMAs) were obtained from formalin-fixed paraffin-embedded samples collected at diagnosis from 3 reactive lymph nodes from 27 Chronic lymphocytic lymphoma/Small Lymphocytic lymphoma (CLL/SLL), 18 Splenic marginal zone lymphoma (SMZL), 22 Burkitt lymphoma (BL) and 44 classical Hodgkin lymphoma (CHL) cases and from 99 consecutive patients affected by DLBCL; the latter were classified in GCB (46 cases) and Non-GCB (53 cases) according to Hans algorithm [33]. All DLBCL cases were treated at our Institution with R-CHOP/CHOP-like regimens. All cases were retrieved from the archives of the Haematopathology Unit, Department of Experimental, Diagnostic and Specialty Medicine - DIMES, University of Bologna and were diagnosed from 2002 to 2011. The protocol was approved by the institutional review board of the University of Bologna. Informed consent was obtained from all patients. TMAs were constructed as previously reported [34]. TMAs sections were investigated by antibodies raised against fixation resistant epitopes of CHK1, p-CHK1 serine (ser) 345, CHK2, p-CHK2 threonine (thr) 68, CDC25, p-CDC25 ser 216, p-H2AX ser 319, c-MYC, BCL2, Ki67 and P53 proteins; the antibody reactivity and sources as well as the antigen retrieval protocols, dilutions and revelation systems are detailed in supplementary Table S4. The cutoff for positivity was set at 30% of cells expressing the protein of interest, unless otherwise specified [34]. For Bcl-2 and c-MYC we applied a cut-off of 70% and 40%, whereas for p53 staining a cut-off of 50% was applied, according to the recent literature [9, 35]. Immunohistochemical preparations were visualized and images were captured using Olympus Dot-slide microscope digital system equipped with the VS110 image analysis software.

### Cell lines and primary cells

The human DLBCL-derived cell lines SUDHL-4, SUDHL-6, the HL cell line KM-H2 and the BL cell line RAMOS were obtained from the German Collection of Microorganisms and Cell Cultures, Department of Human and Animal Cell Cultures (Braunschweig, Germany). The DLBCL derived cell lines (HBL-1, U2932, TMD8, BJAB) were kindly provided by Dr. R.E. Davis (Houston, Tx). Cell lines were cultured in RPMI 1640 medium supplemented with 10% heat-inactivated fetal bovine serum (GIBCO BRL, Gaithersburg, MD), 1% L-glutamine, and penicillin-streptomycin in a humid environment of 5% CO_2_ at 37°C. The phenotypes and genotypes of these cell lines have been previously described [36, 37].

Primary cells from patients were obtained from bone marrow samples, peripheral blood of leukemic phase patients with more than 90% of circulating blasts, neoplastic abdominal and pleural effusions. Normal bone marrow progenitors were harvested from bone marrow aspirations performed in lymphoma patients undergoing initial staging procedures, which then resulted negative for lymphoma infiltration.

### *In vitro* proliferation assay

Cells were seeded in 96-well plates at 50,000 cell/100 μl/well with increasing concentrations of drug (0.005–10 μM) for 24 and 48 hours. Cell viability was assessed by adding WST-1 reagent (Roche Applied Science, Basel, Switzerland) to the culture medium at 1:10 dilution, according to manufacturer's instructions. Viability of primary cells was assessed by using the trypan blue staining, in triplicate experiments.

### Statistical analyses

Survival analyses were performed using the Kaplan-Meier method and differences between groups calculated by using the log-rank test [38]. Multivariate analyses were performed by using the Cox regression model. Procedures to determine the effects of certain conditions on cell proliferation and apoptosis, were performed in 3 independent experiments. The 2-tailed Student *t* test was used to estimate the statistical significance of the differences between results from the 3 experiments. The Fisher's exact test was used to estimate differences in proportions between groups. The Pearson and Spearman's tests were used to establish correlations between different variables. Quantification of protein-band intensities by densitometric analysis was performed using NIH ImageJ software (National Institutes of Health, Bethesda, MD). Hierarchical clustering of immunohistochemical results, displayed in dendrogram in Figure 1A, was generated as reported in supplementary material. The SPSS and PRISM softwares were used for the statistical analyses. Detailed methods are available in supplemental data.

### Western blotting, immunofluorescence and flow cytometry

Preparation of cellular protein lysates, protein quantitation, western immunoblotting and flow cytometry were performed as previously described [39, 40]. A total of 30 μg of protein was denatured in Laemmli buffer at 95°C and separated by SDS-PAGE. Proteins were then electrotransferred onto nitrocellulose membranes and submitted to immunodetection with the relevant antibody. Membrane-bound secondary antibodies (HRP-conjugated goat anti-rabbit or anti-mouse, BioRad) were detected using SuperSignal West Dura Extended Duration Substrate (Pierce Chemical Co., Rockford, IL).

### Immunofluorescence

Cells were seeded on poly-L-lysine coated glass coverslips and incubated overnight at 4°C. Samples were incubated for 1 hour in PBS plus 1% Bovine Serum Albumin (Sigma-Aldrich) to block unspecific binding before incubating with the primary antibody (Cell Signaling) diluted 1:400 in PBS 1% BSA overnight at 4°C. The samples were rinsed in PBS and then incubated with secondary antibody Alexa fluor 568 goat anti-rabbit IgG (Life Technologies) for 40 min at 37°C in darkness. Mounting and nuclei counterstaining were performed using the “pro long antifade reagent with DAPI” (Molecular Probes, Invitrogen) and observed under a fluorescence microscope.

### Flow cytometry

Apoptosis was determined by using the Annexin V–FITC apoptosis detection kit (BD Pharmingen, San Diego, CA) according to the manufacturer's instructions. Data were collected on a FACS Canto II flow cytometer (BD Biosciences, San Jose, CA) using FlowJo software (Tree Star, Ashland, OR).

### Antibodies for western blotting, immunofluorescence and immunohistochemistry

For western blotting, antibodies to the following were purchased from Cell Signaling Technology: p-CHK1 ser 317, p-CHK1 ser 345, CHK1, pCHK2 thr 68, p-H2AX ser 139 (also used for immunofluorescence and immunohistochemistry), p-CDC25c ser 216, CDC25c, p-ATM ser 1981, ATM. C-MYC antibody was purchased from Abcam. For immunohistochemical studies, antibodies were obtained from Novus biologicals (CHK1, pCHK1ser 345, pCDC25 ser 216), Cell Signaling (Danvers, MA) (pCHK2 thr 68, p-H2AX ser 319), Epitomics (Burlingame, CA) (CHK2, CDC25, c-MYC), Menarini (Florence, Italy) (P53).

### Reagents and proliferation assays

The CHK1/2 inhibitors PF-0477736 and AZD-7762 were purchased from Selleckchem (Houston, TX). Doxorubicin was purchased from Sigma Chemicals (Milan, Italy).

### Production of KM-H2 -derived cells with stably inactivated p53

KM-H2 cells stably expressing p53DD, a truncated, dominant-negative form of murine p^53^ [41]and the related empty vector-transduced control cells (pBABE), were obtained as described by Morgenstern JP and Land H [42]. These cell lines were maintained in RPMI supplemented with 10% FBS and selected with puromycin antibiotic (Sigma-Aldrich, Milan, Italy).

### Sanger sequencing of *TP53* and *CDKN2A/B* genes

Detailed information on primer sequences, and Sanger sequencing of *CDKN2A/B* genes are available in supplemental data.

## SUPPLEMENTAL DATA


