# Supplementary material for: Constitutive activation of the DNA damage response pathway as a novel therapeutic target in diffuse large B-cell lymphoma
Source: Oncotarget. 2015 Jan 7;6(9):6553–69. doi: 10.18632/oncotarget.2720 (PMC4466634; doi:10.18632/oncotarget.2720)
Supplement: Supplementary file 1 [file oncotarget-06-6553-s001.pdf]

SUPPLEMENTAL DATA

SUPPLEMENTAL METHODS

Sanger sequencing of *TP53* and *CDKN2A/B* genes

Total cellular RNA was extracted using the RNeasy total RNA isolation kit (Qiagen, Valencia, CA). One microgram of total RNA was reverse transcribed using the M-MLV Reverse Transcriptase (Invitrogen, San Diego, CA). Three overlapping shorter amplicons [amplicon 1 (491 bp): exons 1–5; amplicon 2 (482 bp): exons 5–8; amplicon 3 (498 bp): exons 8–11)] covering the entire coding sequence (GenBank accession number NM_000546.4) were amplified with 2U of FastStart Taq DNA Polymerase (Roche Diagnostics, Mannheim, Germany), 0.8 mM dNTPs, 1 mM MgCl2, and 0.2 M forward and reverse primers (Table S2) in 25 µl reaction volumes. PCR products were purified using QIAquick PCR purification kit (Qiagen) and then directly sequenced using an ABI PRISM 3730 automated DNA sequencer (Applied Biosystems, Foster City, CA) and a Big Dye Terminator DNA sequencing kit (Applied Biosystems). All sequence variations were detected by comparison using the BLAST software tool (www.ncbi.nlm.nih.gov/BLAST/) to reference genome sequence data (GenBank accession number NM_000546.4).

Primer sequences. For each primer pair, the sequence (5'- 3'), the melting temperature TM, the length and the amplicon size are reported. Primers have been designed using Primer3 (v. 0.4.0) Software Tool. P53 F1 [(TGGATTGGCAGCCAGACT), Temperature (T) 60.36 C°, Length 18, Amplicon size 491 base pair (bp)], P53 R1 [(GGGGGTGTGGAATCAACC), T 61.01 C°, length 18, amplicon size 491 bp], P53 F2 [(TCAACAAGATGTTTTGCCAACT), T 59.50 C°, length 22, amplicon size 482 bp], P53 R2 [(GCGGAGATTCTCTTCCTCTGT), T 59.97 C°, length 21, amplicon size 482 bp], P53 F3 [(GGTAATCTACTGGGACGGAACA), T 60.24 C°, length 22, amplicon size 498 bp], P53 R3 [(CTATTGCAAGCAAGGGTTCAA), T 60.25 C°, length 21, amplicon size 498 bp]. For those cell lines with available data in the public domains, the *TP53* status found was in line with the informations available in the Cancer Cell Line Encyclopedia website (http://www.broadinstitute.org/ccle/home) for KM-H2, SUDHL-4 and SUDHL-6, in the IARC TP53 website (http://p53.iarc.fr/CellLines.aspx) for BJAB and RAMOS, and finally in the COSMIC database for U2932 (http://cancer.sanger.ac.uk/cancergenome/projects/cosmic/).

CDKN2A/ARF and *CDKN2B* mutation screening

Genomic re-sequencing of all coding exons of *CDKN2A/ARF* and *CDKN2B* was performed in search of mutations using primers as previously described [1].

For each sample six amplicons were generated using Fast Start Taq DNA Polymerase protocol (Roche, Mannheim, Germany) and AmpliTaq Gold DNA Polymerase LD protocol (Applied Biosystems, Foster City, CA). Amplicons were thereafter purified using QIAquick PCR purification kit (Qiagen) and then directly sequenced using an ABI PRISM 3730 automated DNA sequencer (Applied Biosystems) and a Big Dye Terminator DNA sequencing kit (Applied Biosystems). All sequence variations were detected by comparison using the BLAST software tool (www.ncbi.nlm.nih.gov/BLAST/) to reference genome sequence data (GenBank accession number NM_000077.4, NM_058195.3 e NM_004936, for *CDKN2A*, *ARF*, *CDKN2B*, respectively) obtained from the UCSC browser (http://genome.ucsc.edu/cgi-bin/hgGateway?db = hg18; March 2006 release).

CDKN2A/B deletion analysis

Deletions in *CDKN2A/B* genes were assessed using a Multiplex Ligation-dependent Probe Amplification (MLPA) approach and the SALSA MLPA kit P335 ALL (MRC-Holland) following manufacturer's recommendations (www.mlpa.com).

Bioinformatic analysis of immunohistochemical (IHC) results

To generate hierarchical clustering as displayed in dendrogram in Figure 1A, each antibody (p-CHK1 ser345, p-CHK2 Thr68, p-CDC25c ser216 and p-H2AX ser139) tested immunohistochemically was assigned a binary score value of either 0 or 1 depending on its positivity or negativity, respectively, in all cases studied. Furthermore, a relative frequency score [number of positive cases for a single IHC marker in a specific tumor (DLBCL, BL, cHL, SMZL, CLL) or normal tissue type (reactive lymph nodes) / total number of cases studied of the same tumor or normal tissue type] was calculated for each marker in all tumor subtypes and normal tissues studied. A matrix with the different tumor types and normal tissues types in rows and their IHC scores for the 4 markers in columns was created. Matrix data was analysed using MeV v4.7.4 tool [2]. Hierarchical cluster analysis was performed to classify different tumor types based on IHC marker expression. For every couple of

tumor types and normal tissue type a and b, Euclidean distance D was calculated based on the expression of IHC markers as per the formula (i) $D_{ab} = \sum (a_i - b_i)^2$. The tumor and normal types were furthermore clustered in different groups using a stepwise clustering method. For every step, tumor types or normal tissue type with minimal Euclidean distance were joined together in a cluster and the distance between different clusters was considered taking the maximum distance between 2 different elements each of them belonging to one of the two clusters (complete linkage method).

REFERENCES

1. Iacobucci I, Ferrari A, Lonetti A, et al.CDKN2A/B alterations impair prognosis in adult BCR-ABL1-positive acute lymphoblastic leukemia patients. Clin Cancer Res. 2011; 17:7413–23.
2. Saeed AI, Sharov V, White J, et al. TM4: a free, open-source system for microarray data management and analysis. Biotechniques. 2003; 34:374–378.

SUPPLEMENTARY FIGURES AND TABLES

A

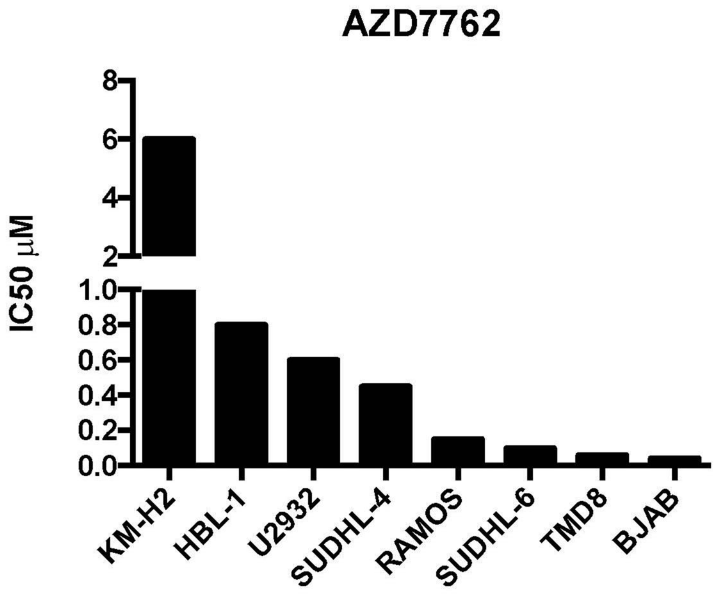

B

Cell line	Cell of origin	PF0477736 IC50 (nM)	AZD7762 IC50 (nM)
BJAB	GCB-DLBCL	9	40
U2932	ABC-DLBCL	160	600
RAMOS	BL	168	150
TMD8	ABC-DLBCL	180	60
SUDHL-6	GCB-DLBCL	200	100
SUDHL-4	GCB-DLBCL	210	450
HBL-1	ABC-DLBCL	230	800
KM-H2	HL	6800	6000

Supplementary Figure S1: Antiproliferative activity of AZD7762 in our panel of B-cell lymphoma cell lines. **(A)** IC50 values after 48 hours of incubation with increasing doses of AZD7762. **(B)** Table showing 48 hours-IC50 values of PF-0477736 and AZD7762 in our panel of B-cell lymphoma cell lines.

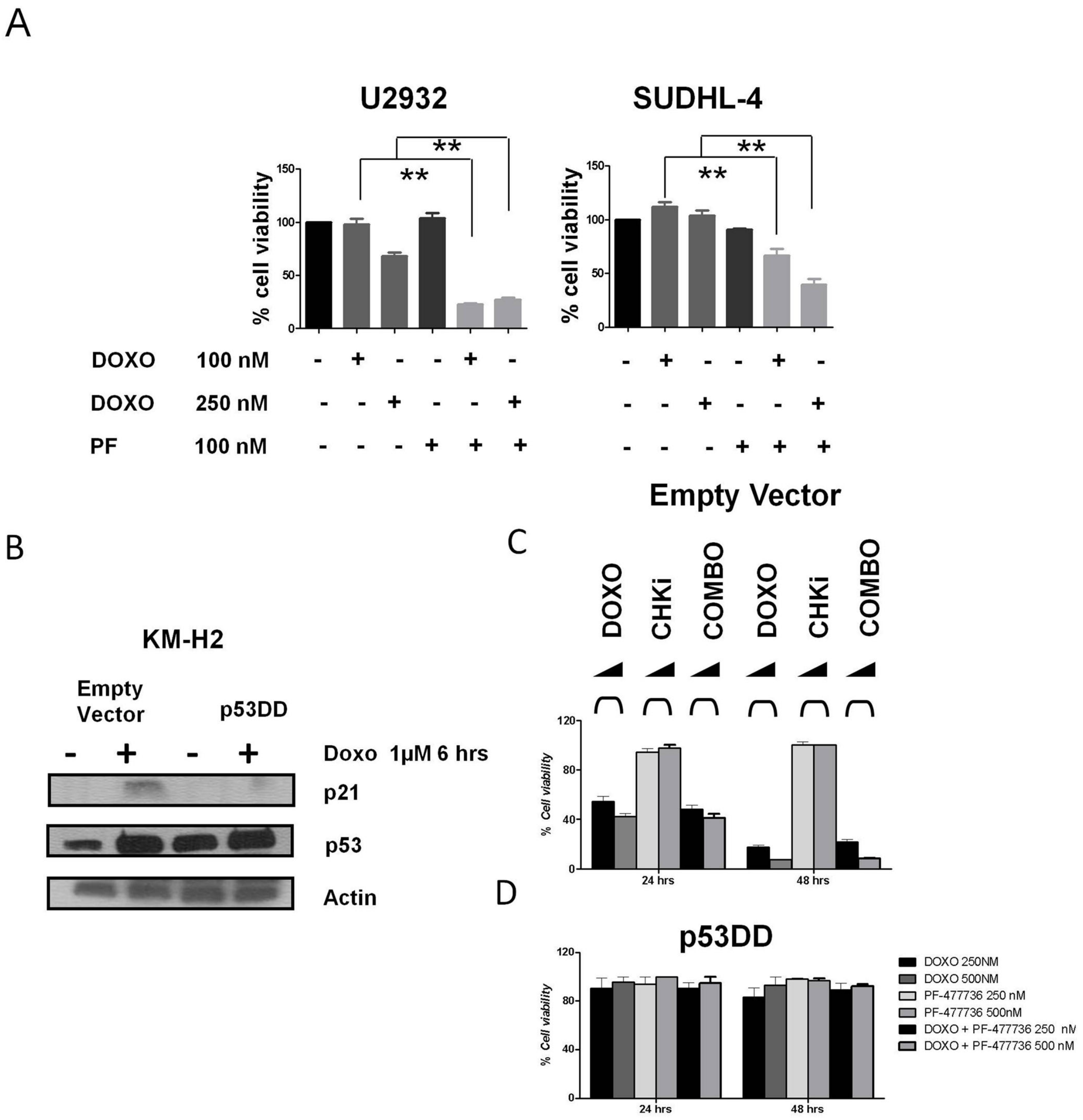

Supplementary Figure S2: P53 status and activity of CHK inhibitors in B-cell lymphoma. (A) PF-0477736 synergizes with Doxorubicin in TP53 mutant DLBCL cells.WST-1 assay showing synergism between PF-0477736 (100 nM) and Doxorubicin (100 and 250 nM) after 48 hours of incubation in SUDHL-4 and U-2932 cells. **(B)** KM-H2 cells were transfected with a retroviral vector expressing the dominant negative *TP53* mutant (p53DD). The stable expression of this functionally inactive p53 mutant resulted in the accumulation of the p53 protein in p53 DD cells. KM-H2 cells transfected with p53DD and treated with Doxorubicin (Doxo) 1 μM for 6 hours lacked p21 expression, compared to empty retroviral vector (pBABE) transfected cells, which retained intact p53 function with normal transcription of p21 following doxorubicin exposure. These results confirm that KM-H2 cells were successfully transfected with the dominant negative p53 mutant p53DD. **(C, D)** KM-H2 cells transfected with empty retroviral vector (pBABE) or p53DD were incubated with doxorubicin (250 and 500 nM), PF-0477736 (250 and 500 nM) and the combination. Cells transfected with the empty vector were sensitive to Doxorubicin, resistant to PF-0477736 and the combination. Although as expected p53DD KM-H2 cells became resistant to Doxorubicin, they remained resistant to PF-0477736 and to the combination, both as 24 and at 48 hours. These findings demonstrate that at least in this cellular context disruption of p53 function is not sufficient to induce sensitivity to CHK inhibition. Each value is the mean of three independent experiments performed in triplicate. $^{*}P < 0.05$; $^{**}P < 0.005$; NS, not significant. Error bars represent s.e.m.

Supplementary Table S1: Patients characteristics and univariate analyses for overall survival

Factor	Patient N°	*P* value
**Gender**		0.75
M	58	
F	41	
**Median Age (range)**	65 (16–87)	**0.002**
< 60 y	37	
≥ 60 y	62	
**Cell of Origin**		0.72
GCB	46	
Non-GCB	53	
**R-CHOP vs R-Other (VNCOP-B/ MACOP-B)**	67 vs 32	0.16
**Ki-67**		0.6
0–90%	66	
≥ 90%	33	
**IPI**		**< 0.001**
0–2	63	
≥3	36	
**p53**		0.65
0–50%	74	
≥ 50%	23 (2 missing [÷])	
**Bcl-2**		0.052
0–70%	28	
≥ 70%	71	
**c-MYC**		0.46
0–40%	49	
≥ 40%	47 (3 missing [§])	
**Bcl2/MYC**		**0.03**
Bcl-2/MYC negative	61	
Bcl-2/MYC positive	35 (3 missing [§])	
**pCHK1/2**		0.06
0–30%	58	
≥ 30%	41	
**pCDC25c**		**0.02**
0–30%	59	
≥ 30%	40	
**γH2AX**		**0.01**

(*Continued*)

Factor	Patient N°	*P* value
0–30%	52	
≥ 30%	47	

÷ Immunohistochemistry data for p53 expression were available for 97 patients; § Immunohistochemistry data for c-MYC expression were available for 96 patients.
Abbreviations: M (male), F (female); VNCOP-B (VP-16, Mitoxantrone, Cyclophosphamide, Vincristine, Prednisone, Bleomycin); MACOP-B (Methotrexate, Doxorubicin, Cyclophosphamide, Vincristine, Prednisone, Bleomycin)., N (number).

Supplementary Table S2: Multivariate analyses for overall survival

Factor	N Patients (n=96)	*P* value	Hazard ratio (95%C.I.)
**Age**		**0.02**	
< 60	36		1
> 60	60		2.97 (1.19–7.35)
**IPI**		**< 0.001**	
0–2	61		1
< 3	35		3.72 (1.82–7.57)
**Bcl-2/MYC**		0.12	
Negative	61		-
Positive	35		
**pCDC25c**		0.26	
Negative	57		-
Positive	39		
**γH2AX**		**0.01**	
Negative	51		1
Positive	45		2.44 (1.19–4.99)

P value was calculated according to the Cox Regression model.

Supplementary Table S3: Detailed results of *TP53* mutations screening in cell lines and primary cells

Cell line name	Disease type	*TP53* mutation	*CDKN2A* status
BJAB	GCB-DLBCL	H193R	WT
SUDHL-4	GCB-DLBCL	R273C	WT
SUDHL-6	GCB-DLBCL	Y234C	WT
HBL-1	ABC-DLBCL	V157A ex5	WT/LOSS
U-2932	ABC-DLBCL	C176Y ex5	WT
TMD8	ABC-DLBCL	WT	LOSS HOMOZYGOUS
RAMOS	BL	I254D ex7	WT
KM-H2	HL	WT	WT
**PATIENT SAMPLES**			
CLL#1	CLL	WT	WT
CLL#2	CLL	WT	WT
CLL#3	CLL	WT	WT
HCL#1	HAIRY CELL LEUKEMIA VARIANT	WT	WT
MZL#1	MARGINAL ZONE LYMPHOMA	WT	WT
DLBCL#1	DLBCL	WT	WT
DLBCL#2	DLBCL	WT	WT
BL#1	BURKITT LYMPHOMA	WT	WT
BL#2	BURKITT LYMPHOMA	R282W	WT
MCL#1	MANTLE CELL LYMPHOMA blastoid variant	WT	WT

Abbreviations: DLBCL (diffuse large B-cell lymphoma), GCB (germinal center), ABC (activated B-cell), BL (Burkitt lymphoma), HL (Hodgkin lymphoma), CLL (chronic B-cell lymphoid leukemia), HCL (Hairy cell leukemia), MZL (Marginal zone lymphoma), MCL (Mantle cell lymphoma), WT (wild type).

Supplementary Table S4: Detailed informations on antibodies and conditions used for the immunohistochemistry study

Molecule	Antibody type	Source	Dilution	Retrieval	Revelation system
CHK1	Rabbit polyclonal	NOVUS : NB100–91696	1:20	PtLink, low pH 97°C x 20’	K8012*
pCHK1 (ser 345)	Rabbit polyclonal	NOVUS: NBPI-60799	1:20	PtLink, low pH 97°C x 20’	K8012
CHK2	Rabbit monoclonal	EPITOMICS: 3428–1	1:400	PtLink, high pH 92°C x 5’	K5005§
pCHK2 (thr 68)	Rabbit polyclonal	CELL SIGNALING: 2661	1:200	PtLink, high pH 92°C x 5’	K5005
CDC25	Rabbit monoclonal	EPITOMICS: 1302–1	1:9600	PtLink, high pH 92°C x 5’	K5005
pCDC25 (ser 216)	Rabbit polyclonal	NOVUS: NB100–92494	1:20	PC+MW900W X 3’+TRIS EDTA	K8012
γH2AX (ser 139)	Rabbit polyclonal	CELL SIGNALING: 2577	1:25	PtLink, high pH 92°C x 5’	K5005
c-MYC	Rabbit monoclonal	EPITOMICS: 1472	1:100	PtLink, high pH 92°C x 5’	K5005
P53	Mouse monoclonal	MENARINI: NCL-P53	1:200	PtLink, high pH 92°C x 5’	K5005

Supplementary table 4. Immunohistochemistry: antibodies, antigen retrieval protocols, dilutions and revelation systems. *K8012: Envision Flex, (Dako Cytomation, Denmark); §K5005: Real Detection System Alkaline Phosphatase/RED rabbit/mouse (Dako Cytomation, Denmark).
